# Comparison of bone reamer and trephine for foraminoplasty in percutaneous endoscopic lumbar discectomy based on 3D slicer and Digimizer software

**DOI:** 10.1186/s13018-023-04270-x

**Published:** 2024-01-12

**Authors:** Jiewei Sun, Jun Wang, Ruiji Wu, Zhi Zhao, Bingkai Fan, Jie Cai, Fabo Feng

**Affiliations:** 1 Cardiothoracic Surgery Department, The First People’s Hospital of Fuyang, No. 429, Beihuan Road, Fuchun Street, Fuyang District, Hangzhou, 311400 Zhejiang China; 2 The orthopaedic, Xiaoshan Hospital Affiliated to Wenzhou Medical University, Hangzhou, Zhejiang China; 3 Second Affiliated Hospital of Zhejiang Chinese Medical University, Hangzhou, 310005 Zhejiang China; 4 Center for Plastic and Reconstructive Surgery, Department of Orthopedics, Zhejiang Provincial People’s Hospital (Affiliated People’s Hospital, Hangzhou Medical College), Hangzhou, Zhejiang China

**Keywords:** Foraminoplasty, 3D slicer, Digimizer, Bone reamer, Trephine

## Abstract

**Objective:**

To explore the applicability of bone reamer and trephine for foraminoscopy in percutaneous endoscopic lumbar discectomy (PELD), and to provide a theoretical basis for foraminoplasty options in clinical practice.

**Methods:**

This study was a prospective cohort study. Sixty-three consecutive patients who underwent PELD for lumbar disc herniation between May 2021 and July 2022 were analysed. Foraminoplasty were performed by bone reamer or trephine. The amount of bone removed and the foramen area enlarged during foraminoplasty by both tools were measured by 3D slicer and Digimizer software, and the numbers of fluoroscopic views were recorded.

**Results:**

The bone reamer removed less bone in the Superior Articular Process (SAP) than the trephine (*t* = 17.507, *P* < 0.001), and the area enlarged by the bone reamer was smaller than that of the trephine (*t* = 10.042, *P* = 0.002). The overall numbers of fluoroscopic views were significantly more in the bone reamer group than in the trephine group (*t* = 19.003, *P* < 0.001). In the bone reamer group, when the area of preoperative (FPZ) was no less than 54.55 mm^2^, the mean number of fluoroscopic views significantly decreased (*t* = 14.443, *P* = 0.001).

**Conclusion:**

Bone reamer was safer and trephine was more efficient for foraminoscopy in PELD. An area of preoperative (FPZ) of 54.55 mm^2^ can be used as a critical value: bone reamer reduced the risk for cases above the value, while trephine improved the efficiency for cases less than the value.

## Introduction

Transforaminal percutaneous endoscopic lumbar discectomy (PELD) has become a widely used technique for Lumbar Disc Herniation (LDH) with advantages including shorter hospitalstay, quicker postoperative rehabilitation and restored spinal stability. PELD requires the enlargement of the target foramina, the so-called foraminoplasty, in order to place a working cannula through the intervertebral foramen. Hence, a secure, efficient, and rapid foraminoplasty is a key step of PELD, especially the Transforaminal Endoscopic Spine System (TESSYS) technique [1]. Two mainly used tools to perform foraminoplasty including a fluoroscopy-guided bone reamer or trephine have mainly described in previous studies. Bone reamer is less likely to destabilize the facet joint and cause the neural injury [3], but the time-demanding procedure increases the risk of radiation exposure [4]; Trephine is more efficient [5] and less radiation exposure [6], but it tends to excise too much bone around the intervertebral foramen [7], increasing the risk of lumbar instability and neural injury [8]. Therefore, it is important to find a balance between the dose of X-ray exposure and the amount of bone removed while ensuring the effectiveness of foraminoplasty.

The cross-sectional area of the endoscopic device is generally circular with a diameter of 7.5 mm [9], while there are significant differences in the size of the intervertebral foramen [10].Hence, the degrees of foraminoplasty required are individual. Bone reamer can be used to guarantee the security when the foramen is large and only mild foraminoplasty is required, while trephine is more suitable to improve efficiency as well as to reduce radiation exposure when the foramen is stenosed and a high degree of foraminoplasty is required. However, the choice of foraminoplasty tools in clinical practice is mostly based on the operator's proficiency instead of the characteristics of the intervertebral foramen and the degree of foraminoplasty required.

In this study, the preoperative and postoperative area of foraminoplasty zone (FPZ) will be measured by 3D slicer and Digimizer software, while the amount of surrounding bone loss as well as intraoperative radiation exposure will be compared to find a balance between the dose of radiation exposure and bone resection, aiming to provide a theoretical basis for the selection of foraminoplasty tools in clinical practice.

## Data and methods

### General data

Sixty-three consecutive patients who underwent PELD for LDH between May 2021 and July 2022 were analysed. A total of 63 cases were included, and the LDH was at the L4/5 or L5/S1 segments. Foraminoplasty were performed by bone reamer or trephine. We first selected patients suitable for surgery based on inclusion and exclusion criteria, and then grouped them randomly by computer. The study was carried out in accordance with the ethical standards of the Declaration of Helsinki of the World Medical Association and was approved by our institutional review board.

### Inclusion and exclusion criteria

Inclusion criteria: (1) clear diagnosis of LDH; (2) single-segment LDH; (3) unilateral symptoms; (4) cases of L5/S1 segment without high iliac crest; (5) intact imaging data; (6) without previous lumbar spine surgery history; (7) without associated anatomical disorientation (e.g. Spondylolisthesis, Scoliosis etc.).

Exclusion criteria: (1) tandem spine stenosis; (2) LDH more than one segment; (3) bilateral symptoms; (4) cases of L5/S1 segment with high iliac crest; (5) absence of imaging data. (6) Previous history of lumbar spine surgery; (7) with associated anatomical disorientation (e.g. Spondylolisthesis, Scoliosis etc.).

### Surgical methods

PELD and standard TESSYS technique was performed in both groups.

**Fig. 1:**
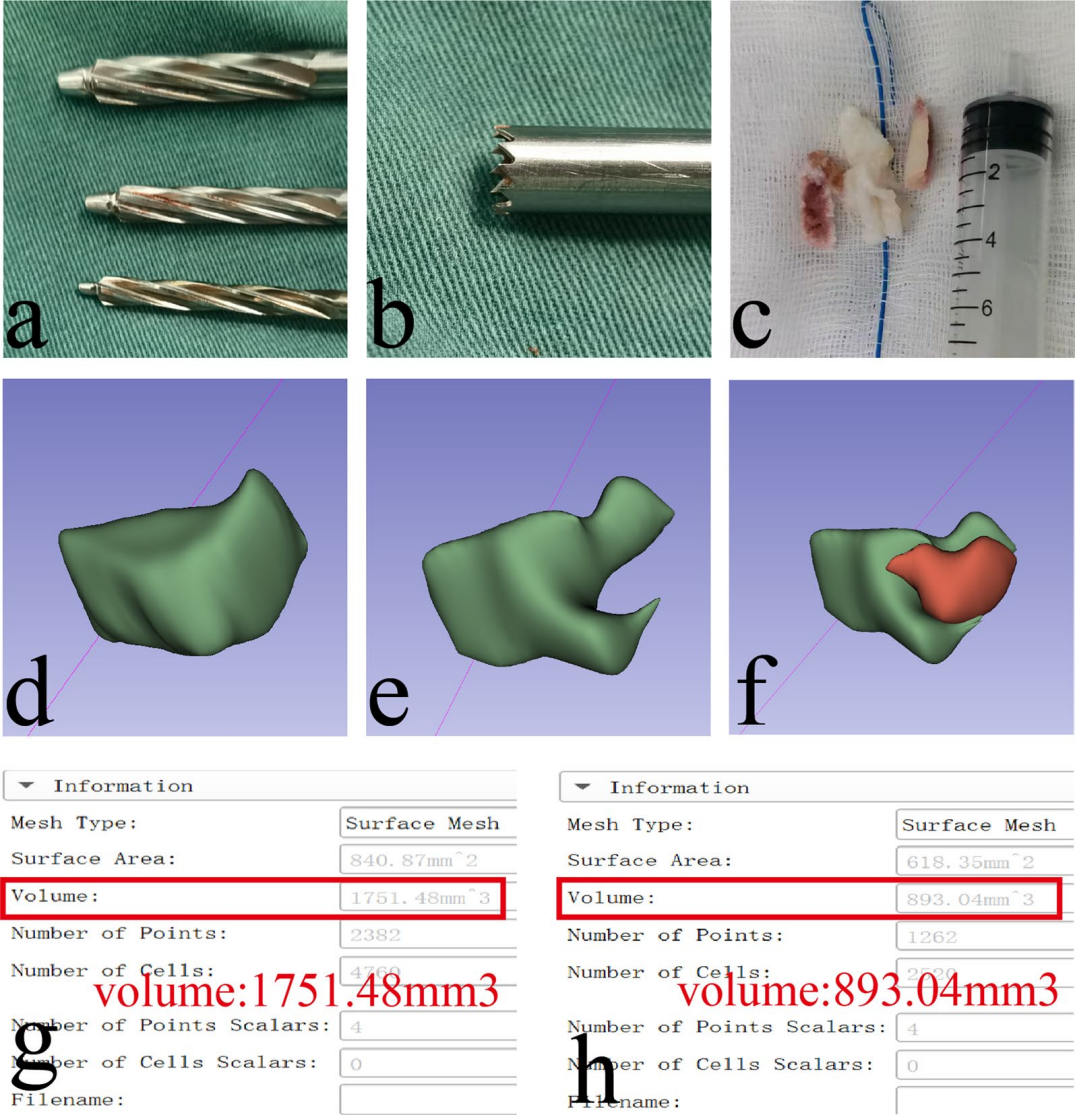
**a**: Three levels of bone reamers with a diameter of 4.6, 5.9 and 7.5 mm respectively. **b**: A trephine with a diameter of 7.5 mm; **c**: Bone removed by trephine. **d**: Preoperative modeling of SAP; **e**: Postoperative modeling of SAP; **f**: The red part was the removed bone mass of SAP; **g**: Preoperative volume of SAP. **h**: Postoperative volume of SAP

Bone reamer group: Step by step foraminoplasty was carried out with three levels of bone reamers with a diameter of 4.6, 5.9 and 7.5 mm (Shandong Guanlong Medical Products Co., Ltd) respectively, (Fig. 1-a). The foraminoplasty were accomplished when the fluoroscopy showed that the head of the bone reamer was located between the line of the medial edge of the pedicle and the line of the spinous processes on the anteroposterior view, and it was located at the posterior-superior corner of the inferior vertebral body on the lateral view.

(2) Trephine group: A trephine (Shandong Guanlong Medical Products Co., Ltd) with a diameter of 7.5 mm was used for once foraminoplasty, (Fig. 1, b-c). The foraminoplasty were completed when the fluoroscopy showed that the head of the trephine was located between the line of the medial edge of the pedicle and the line of spinous processes on the anteroposterior view, and it was located at the posterior-superior corner of the inferior vertebral body on the lateral view.

## Study method

### Calculation of bone loss using 3D slicer software modeling

The amount of bone removed by foraminoplasty was mainly from the Superior Articular Process (SAP), so we merely modeled the preoperative and postoperative SAP.

1. Preoperative modeling: ① imported the preoperative lumbar spine CT data into 3D Slicer software in DICOM format and adjusted the image size appropriately; ② added a new mask in the Segment Editor board and used the paint tool to depict the coverage of SAP from the tip to the basal, until the SAP moves into the transverse process; ③ observed the 3D model in the show 3D interface. ④The 3D model was exported in model format under the Segmentations module, and then the volume size of the model can be read directly in the Models panel. The unit of model volume was mm^3^, and the results were kept in 2 decimal places. (Fig. 1d and g).
2. Postoperative modeling: the method and the number of layers modeled were the same as preoperative modeling. For the rare instances when the tips were resected, the bottom coverage of the postoperative model was ensured to be the same as that of the preoperative model. (Fig. 1e, f and h).

### Measurement of the area of preoperative and postoperative FPZ

The lumbar intervertebral foramen is irregular oval. Combining the relevant literature and our clinical experience, we believed that the main performing zone for foraminoplasty was the area below the line connecting the posterior inferior edge of the superior vertebral body with the tip of the SAP. Therefore, to quantify the area of the foraminoplasty zone, we defined the lumbar FPZ as an irregular quadrilateral with four sides: the height of the posterior edge of the intervertebral space (h), the distance from the posterior superior edge of the inferior vertebral body to the lowest point of the vertebral arch (b), the distance from the lowest point of the vertebral arch to the tip of the SAP (c), and the distance from the tip of the SAP to the posterior inferior edge of the superior vertebral body (a), and the area of the quadrilateral (s) was equivalent to that of the foraminoplasty zone.

**Fig. 2:**
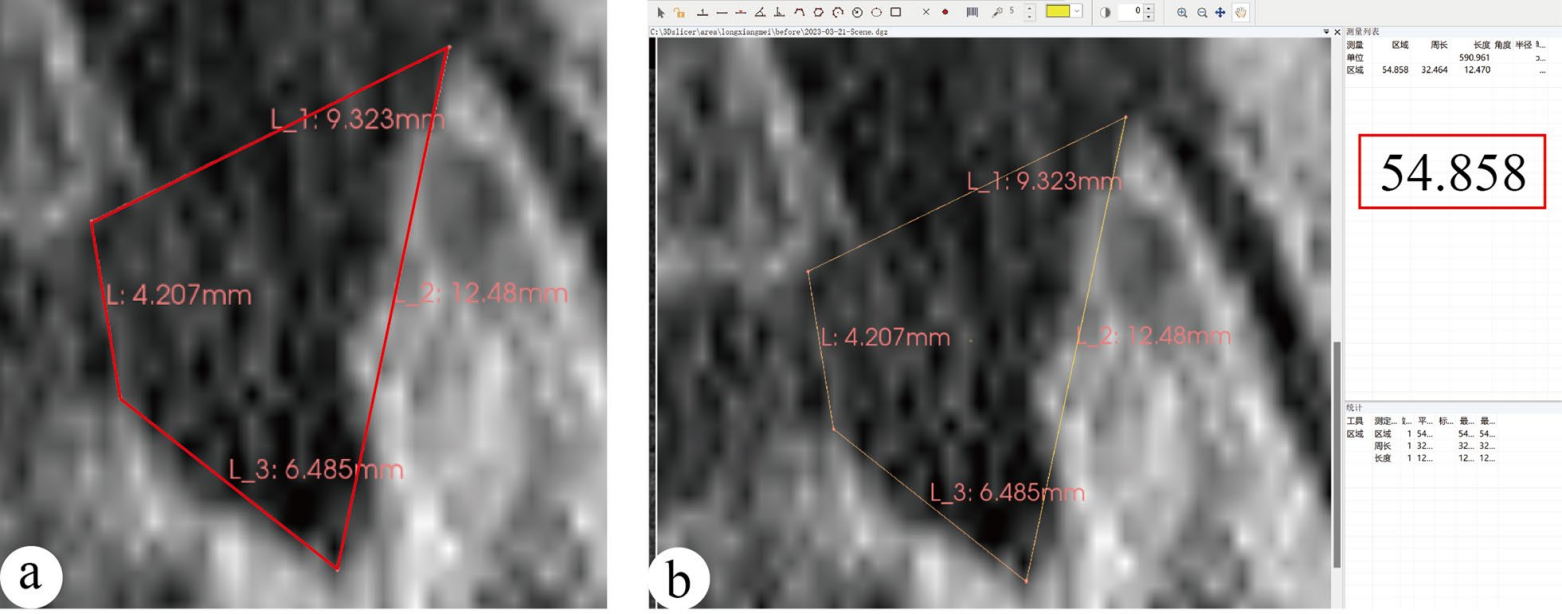
**a** The length of each boundary of the FPZ was measured by 3D slicer software. **b** The area of the FPZ was measured by digimizer software

**Fig. 3:**
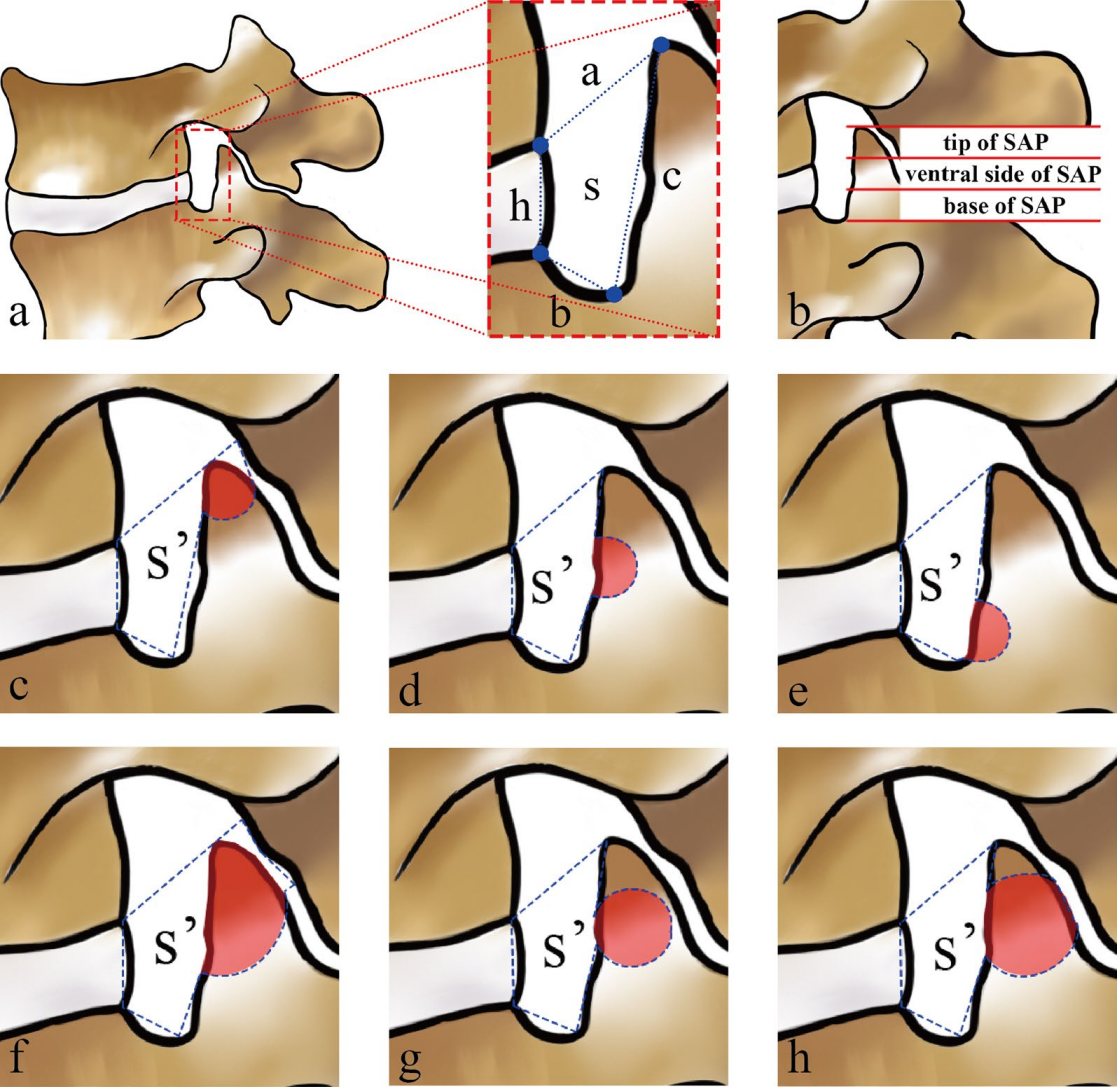
**a**: Schematic diagram of the boundary and area measurement of the FPZ; **b**: Schematic diagram of the division of the SAP into three parts; **c**: Area measurement method of the FPZ after the tip of the SAP was resected; **d**: Area measurement method of the FPZ after the ventral side of the SAP was resected; **e**: Area measurement method of the FPZ after the base of the SAP was resected; **f–h**: Area measurement method of the FPZ after multiple parts were resected

1. Preoperative measurements: ① imported the transverse CT image into 3D slicer software in DICOM format, the software will automatically reconstruct the function to generate sagittal plane images; ② The sagittal image was adjusted to the foraminal level; ③ used the Line tool to measure the height of the posterior edge of the intervertebral space as (h); the height of the posterior superior edge of the inferior vertebral body to the lowest point of the inferior vertebral arch (b); the lowest point of the inferior vertebral arch to the tip of the SAP was (c); the tip of the SAP to the posterior inferior edge of the superior vertebral body was (a). (Fig. 2-a); ④imported the images containing the above four lengths into Digimizer software in JPG format, after setting the unit length, use the area measurement tool to connect the four sides of FPZ in turn, measure the area of FPZ. (Fig. 2-b).
2. Postoperative measurements: The measurement methods were basically the same as those preoperative. As the intraoperative foraminoplasty would resect part of the SAP (Fig. 3-a-b)and enlarge the FPZ to different degrees, we formulated the boundaries of the FPZ postoperatively as follows: ① The tip of the SAP was removed (Fig. 3-c): the posterior inferior edge of the superior vertebral body was defined as the starting point, and the tip of the original SAP was attached and extended to intersect with the inferior articular process Then the boundary of the foraminoplasty zone went along the bony surface of the inferior articular process and the SAP to the lowest point of the vertebral arch as well as the posterior superior edge of the inferior vertebral body, and ended at the posterior inferior edge of the superior vertebral body. ②The ventral side of the SAP was removed (Fig. 3-d): the removed part was included in the range of the foraminoplasty zone. Except for the arc extension of the line from the tip of the superior articular process to the lowest point of the pedicle, the rest of the lines remained unchanged. \
3. The base of the SAP was removed (Fig. 3-e): the resected portion is also included in the foraminoplasty zone. Removal of the basal portion resulted in an arcuate lengthening of the line from the tip of the SAP to the lowest point of the vertebral arch and the line from the lowest point of the vertebral arch to the posterior superior edge of the inferior vertebral body, while the rest of the lines remained unchanged.
4. The SAP was removed in multiple parts (Fig. 3-f–h): all the resected parts were included in the foraminoplasty zone. The upper boundary of the foraminoplasty zone was the line connecting the posterior inferior edge of the superior vertebral body to the tip of the preoperative SAP or its extension. The rest of the boundary was determined according to the resection degree, and generally the height of the posterior edge of the intervertebral space (h) remained unchanged.

### Recording of the dose of radiation exposure

From the first time that the foraminoplasty tool (bone reamer or trephine) contacting with SAP to the complete withdrawal of the foraminoplasty tool from the foramen, the number of anteroposterior views and lateral views of fluoroscopy were counted as a single sheet, and the radiation exposure dose was the sum of all single sheets.

### Statistical methods

SPSS 25.0 software was used for statistical analysis, and quantitative data were presented as mean ± standard deviation. T-test was used for comparison of measurement data between groups, whlie rank sum test and chi-square test were used for comparison of radiation exposure between groups. The difference was considered statistically significant if *P* < 0.05.

## Results

### General information

A total of 63 cases were enrolled in this study, Group-R(bone reamer) consisted of 31 cases, Group-T(trephine) consisted of 32 cases, of which 34 were male and 29 were female. The minimum age was 23 years old, the maximum age was 85 years old, and the average age was 50 years old. No signifificant variation in sex (*X*^2^ = 0.765, *P* = 0.382) and age (*t* = 0.595, *P* = 0.444) distribution was observed between the two groups. There were no statistical difference in the symptom composition ratio (*X*^2^ = 4.429, *P* = 0.513), while a slight difference in the segment composition ratio (*X*^2^ = 4.598, *P* = 0.032), (Table 1).

**Table 1:** General information of the two groups

Indicators	Group-R (*n* = 31)	Group-T (*n* = 32)	t/X^2^	*P* value
Age (year)	48.23 ± 15.78	50.84 ± 17.53	0.595	0.444
*Gender*				
Male	15	19		
Female	16	13	0.765	0.382
*Segmental*				
L_4_/L_5_	20	12		
L_5_/S_1_	11	20	4.598	0.032
*Segmental*				
Left	19	17		
Right	12	15	4.429	0.513

Bone reamer resected significantly less bone of the SAP than trephine (*t* = 17.507, *P* = 0.001), (Table 2).

**Table 2:** Differences in the volume of the SAP between the two groups pre- and postoperatively

Indicators	Group-R (*n* = 31)	Group-T (*n* = 32)	*T* value	*P* value
Preoperativ SAP zone volume (mm^3^)	1269. 62 ± 180.15	1354.48 ± 369.50	6.077	0.017
Postoperative SAP zone volume (mm^3^)	1096.40 ± 165.27	805.16 ± 284.97	4.440	0.037
Excision of the SAP zone volume (mm^3^)	173.22 ± 88.19	549.31 ± 231.43	17.507	0.001
Percentage of resection (%)	13.56 ± 6.57	40.83 ± 14.20	12.387	0.001

The area of FPZ expanded by the bone reamer was smaller than that of trephine (*t* = 10.042, *p* = 0.002), (Table 3).

**Table 3:** Difference in the area of the FPZ between the two groups pre- and postoperatively

Indicators	Group-R (*n* = 31)	Group-T (*n* = 32)	*T* value	*P* value
Area of preoperative FPZ (mm^2^)	53.17 ± 12.86	54.69 ± 16.60	1.960	0.167
Area of postoperative FPZ (mm^2^)	66.13 ± 16.47	81.98 ± 22.35	1.987	0.164
Expanded area (mm^2^)	12.96 ± 11.41	27.30 ± 21.48	10.042	0.002

**Fig. 4:**
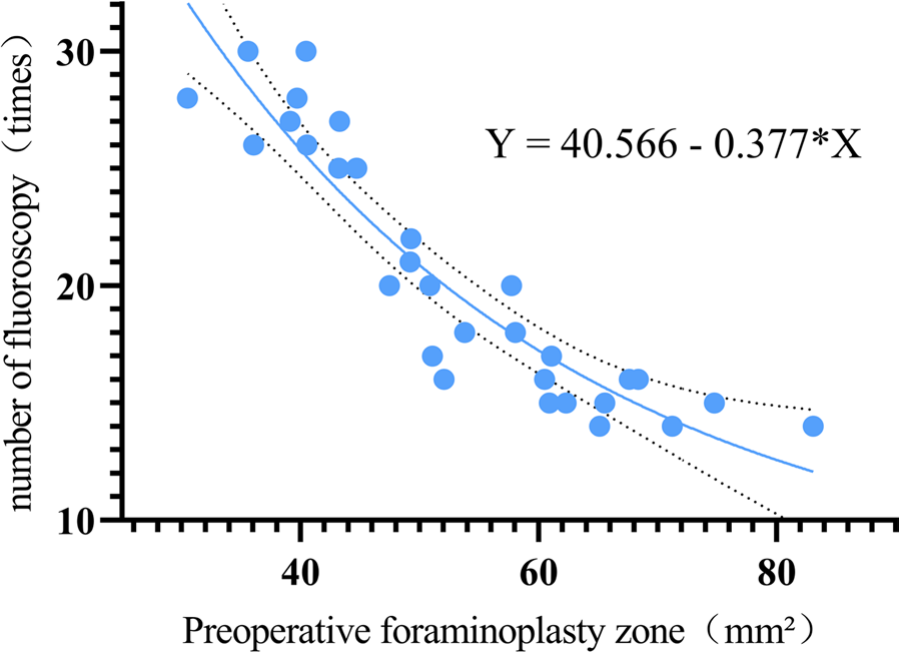
Fitted curves of 31 cases in the bone drilling group

The overall number of fluoroscopy was significantly higher in the Group-R than the Group-T (*t* = 19.003, *P* < 0.001). Linear fit of the data from both groups revealed that there was a significant correlation between the number of fluoroscopy and the area of the preoperative FPZ in the Group-R, and there was a obvious inflection point at 20 fluoroscopic views. Therefore, we analysed the relationship between the number of fluoroscopic views and the area of preoperative FPZ in the Group-R by regression equation: number of fluoroscopy = 40.566–0.377*area of preoperative FPZ (Fig. 3). When the number of fluoroscopy was 20, the area of preoperative FPZ was 54.55 mm^2^. In the Group-R, when the area of preoperative FPZ was no less than 54.55 mm^2^, the mean number of fluoroscopy significantly decreased. (*t* = 14.443, *P* = 0.001)(Table 4), (Fig. 4).

**Table 4:** Relationship between the number of fluoroscopy and the area of the FPZ

Indicators	Group-R	Group-T	*T* value	*P* value
Overall number of fluoroscopy	20.52 ± 5.38	13.25 ± 3.06	19.003	0.001
Preoperative FPZ ≥ 54.55mm^2^	15.77 ± 1.74	11.64 ± 2.41	0.738	0.398
Preoperative FPZ < 54.55mm^2^	23.94 ± 4.39	14.50 ± 2.98	88.337	0.010
*T* value	14.443	0.571		
*P* value	0.001	0.456

### Comparison of adverse events between the Group-R and the Group-T

**Fig. 5:**
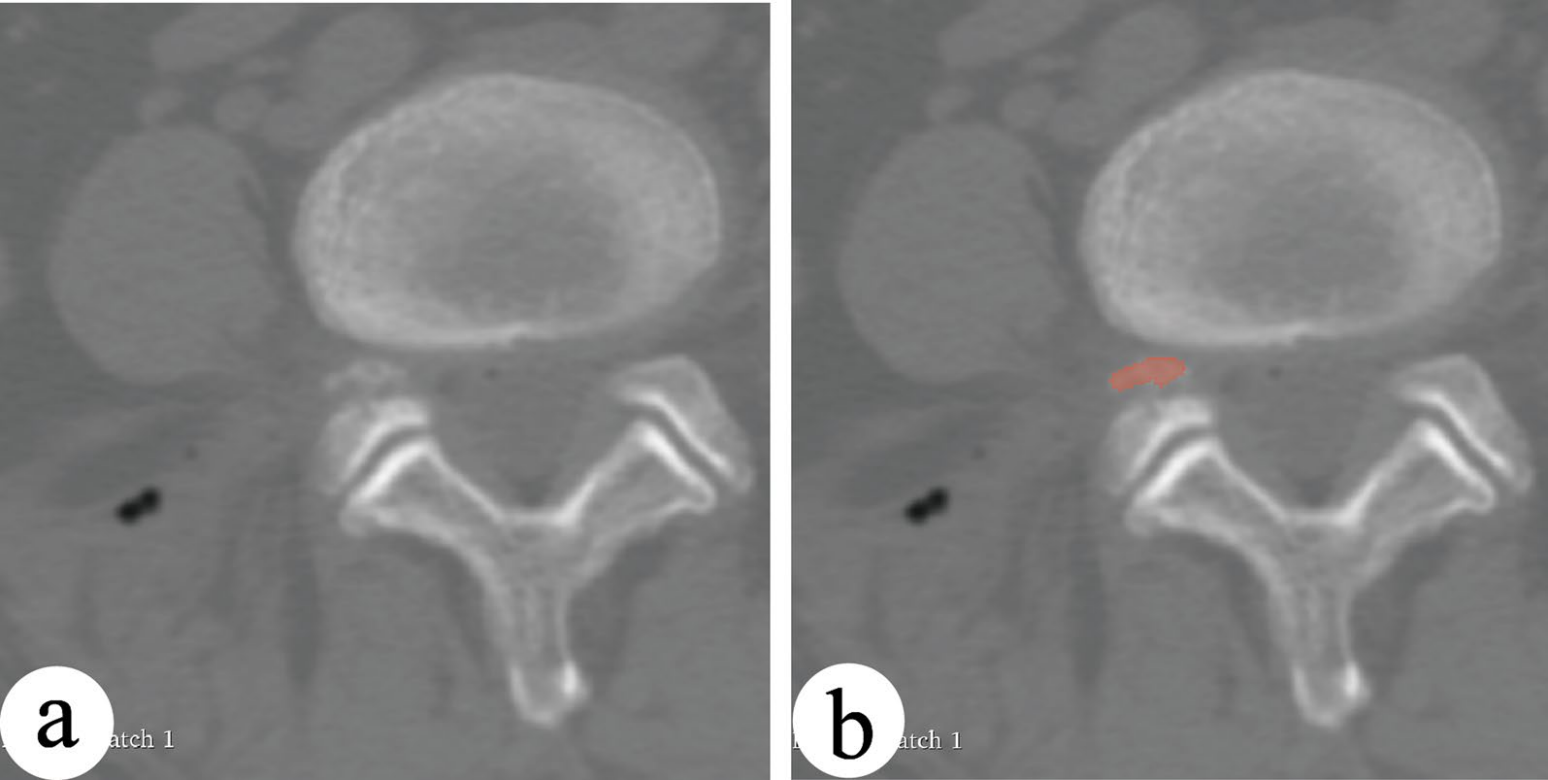
**a** One case of bone fragment falling into the lateral recess in the bone reamer group. **b** The red part is the bone fragment

In the Group-R, there was a complication of bone fragment falling into the lateral recess in one case (Fig. 5). Then we performed a laminar approach revision surgery and the postoperative recovery was acceptable. No significant adverse events were found in the Group-T.

## Discussion

Foraminoplasty is an important step in PELD, especially the TESSYS technique [11]. The amount of bone resection and X-ray exposure during the procedure is closely related to the selection of the foraminoplasty tool [12]. The bone reamer and the trephine, as commonly used foraminoplasty instruments in current clinical practice [13], have their own advantages and disadvantages. The bone reamer restored more bone mass [14], but were low-efficiency and led to more X-ray exposure. The trephine was efficient [15], while the degree of bone resection was higher and might lead to lumbar instability [16]. Therefore, this study intended to find an equilibrium between the amount of X-ray exposure and bone resection, aiming to provide a theoretical basis for the selection of foraminoplasty tools in clinical practice.

As to the bone resection volume, the Group-R (173.22 ± 88.19 mm^3^) was significantly less than the Group-T (549.31 ± 231.43 mm^3^). There may be two reasons for this discrepancy: (i) the bone reamer grinded away the bone through the frictional force generated by rotating the front outer drill teeth, so the amount of bone excised was limited [17]. (ii) The trephine removed bone through the shearing force generated by rotating the frontannular teeth, and it was capable of resecting all the bone mass within the range of the annular teeth. In addition, the Group-R (13.56 ± 6.57%) destrcted less of SAP than the Group-T (40.83 ± 14.20%) according to the percentage of resection The SAP is an important component of the lumbar stabilization system, and more than 30% [8] of the SAP resected increases the risk of instability [18], [19]. Therefore, in accordance with the comparative results of the amount of bone destruction, the bone reamer had less negative impact on lumbar spine stability than the trephine and was more suitable to be selected as a foraminoplasty instrument.

Foraminoplasty in both groups was all successfully achieved. Regarding the enlarged FPZ, the Group-R (12.96 ± 11.41 mm^2^) was slightly less than the Group-T (27.30 ± 21.48 mm^2^), consistent with the results of the amount of bone resection. The results revealed that the bone reamer destroyed less bone in the SAP, and the enlarged FPZ would be relatively limited.

As to the comparison of X-ray exposure, the Group-R (20.52 ± 5.38 times) was significantly more than the Group-T (13.25 ± 3.06 times). The bone reamer used for foraminoplasty in this study was graded, which would naturally be less efficient than a single-shape trephine. Currently most of bone reamers the clinical used for foraminoplastywas step-by-step in clinical practice, because surgeons needed to insert the head end of bone reamer deep into the foramen in order to effectively perform the foraminoplasty [20]. If the 7.5 mm bone reamer was used directly for foraminoplasty, the head end of the bone reamer was often unable to enter the foramen directly. Rotation happened immediately and cause the bone reamer to slip, which would change the direction of foraminoplasty. In contrast, the head end of the trephine can anchor on the SAP due to the presence of annular serrations [21], which avoided slippage during rotation. In addition, the amount of X-ray exposure was almost proportional to the foraminoplasty time, and an increase in the number of fluoroscopic views predicted a longer foraminoplast time, which added physiological and psychological stress on the patient. Therefore, the results from the amount of X-ray exposure indicated that bone reamer molding was less efficient than trephine.

To further investigate the relevant factors affecting the foraminoplasty efficiency, we performed correlation analysis with linear fitting on all data. In the Group-R, there was a significant correlation between the number of fluoroscopic views and the preoperative area of FPZ and there was a significant inflection point in the curve when the number of fluoroscopic views were 20. The regression equation yielded that number of fluoroscopic views = 40.566–0.377*area of preoperative FPZ, and the area of preoperative FPZ was 54.55 mm^2^ when the number of fluoroscopic views was 20. Therefore, we believed that when the area of preoperative FPZ was no less than 54.55 mm^2^, the head end of the bone reamer with a diameter of 7.5 mm can enter the foramen, and the head end of the bone reamer was not prone to rotate. In contrast, when the preoperative molding area was less than 54.55 mm^2^, the head end of the bone reamer with a diameter of 7.5 mm may be unable to enter the foramen smoothly. There was also the possibility of relative slippage, which made it necessary to increase the number of fluoroscopic views during the process to make sure that the direction of the head end of the bone reamer was correct before continuing the foraminoplasty, thus increasing the number of fluoroscopic views and prolonging the molding time. In addition, after reviewing the case of bone fragment falling into the lateral recess in the Group-R, we concluded that the patient was an elderly woman and the preoperative molding area was 43.22 mm^2^, which was less than 54.55 mm^2^, so the head end of the 7.5 mm bone reamer did not enter the foramen during foraminoplasty. When the bone reamer rotated, the head end slided subsequently. Moreover, the patient had risk factors of osteoporosis, and the bone mass of the SAP was loose. As a result, the fragmented bone fell into the lateral recess under the push force of the head end of the bone reamer and compressed the exiting nerve root.

However, this study also has the following limitations: first, the sample size was small with 63 cases included in this study; Second, the boundary and boundaries of the molding zone were determinated on the sagittal plane, and there was usually a certain dorsal offset in the puncture angle of the foraminoplasty tool [22], thus the cross-section of the intervertebral foramen in contact with the foraminoplasty tool may tend to be more elliptical. Additionally, not always 3D slicer and Digimizer might be available for adequate measurement of the foraminal area in all medical centers, this can be a problem when trying to measure FPZ area. The above deficiencies are also the next direction we will consider.

## Conclusion

Bone reamer was safer and trephine was more efficient for foraminoplasty in PELD. When the preoperative FPZ was no less than 54.55 mm^2^, bone reamer was safer for foraminoplasty. In contrast, trephine was more efficient when the preoperative FPZ was less than 54.55 mm^2^.
